# Successful Pregnancy and Fetal Outcomes Following Brentuximab Vedotin for Early Relapsed Classic Hodgkin Lymphoma After Autologous Stem Cell Transplant

**DOI:** 10.7759/cureus.57291

**Published:** 2024-03-30

**Authors:** Akari Goto, Chisa Fujita, Hiroto Horiguchi, Satoshi Iyama, Masayoshi Kobune

**Affiliations:** 1 Department of Hematology, Sapporo Medical University School of Medicine, Sapporo, JPN

**Keywords:** classic hodgkin lymphoma, maternal and fetal outcome, delivery, fetal outcome, autologous stem cell transplant, pregnancy, fertility, relapsed-refractory hodgkin lymphoma, adolescent and young adult, brentuximab vedotin

## Abstract

Brentuximab vedotin (BV), an anti-CD30 antibody with monomethyl auristatin E conjugate, has shown clinical effects against relapsed/refractory classic Hodgkin lymphoma (cHL) and hence is widely used in the clinical setting. We report a special clinical case of successful pregnancy and fetal outcome in a patient with cHL who achieved long-term remission with BV for early relapse after an autologous stem cell transplant (auto-SCT). A 27-year-old woman with advanced cHL achieved complete response (CR) after six cycles of doxorubicin, bleomycin, vinblastine, and dacarbazine (ABVD) regimen. Embryos obtained from intracytoplasmic sperm injection were cryopreserved before the initiation of induction chemotherapy. Despite achieving a second CR following intensive salvage chemotherapy, auto-SCT, and radiotherapy, she relapsed again six months after transplantation. BV monotherapy was administered as salvage therapy. She completed 16 cycles of BV and achieved CR. Six months after BV completion, she expressed her desire to bear a child. She achieved pregnancy through third in vitro fertilization and embryo transfer and delivered a healthy baby. BV may provide a potentially curative treatment for patients with cHL relapsed after auto-SCT. Pregnancy should be avoided during BV administration up to a certain period after the end of administration. Fertility preservation is important for adolescent and young adult cancer survivors, and patients should be informed of cancer-related infertility and fertility preservation options prior to the initiation of cancer treatment.

## Introduction

Classic Hodgkin lymphoma (cHL) is histologically characterized by the presence of large mononuclear Hodgkin cells and multinucleated Reed-Sternberg cells surrounded by an inflammatory microenvironment. Adolescents and young adults, defined as those aged 15-39 years [1], comprise the largest age group affected by cHL. Over the last four decades, the prognosis of cHL has improved with the advancement of novel treatment strategies, resulting in high cure rates, with a greater than 90% five-year overall survival rate for early-stage patients [2]. The standard care for patients with relapsed or primary refractory cHL, defined by progression during chemotherapy or relapse after induction chemotherapy, is high-dose chemotherapy followed by autologous stem cell transplant (auto-SCT); however, data from a number of studies suggest that up to 50% of patients with relapsed/refractory (R/R) cHL will ultimately relapse after auto-SCT [3]. The cHL patients who relapse after auto-SCT have traditionally poor outcomes with a median overall survival (OS) of one or two years, and particularly, poor outcomes are experienced by those with early relapse, defined as relapse within 12 months after auto-SCT [4,5].

Brentuximab vedotin (BV) is an anti-CD30 antibody conjugated by a protease-cleavable linker to a microtubule-disrupting agent, monomethyl auristatin E (MMAE). In a phase Ⅱ study of BV in patients with R/R cHL after failed auto-SCT, five-year follow-up data demonstrated that a subset of patients (9% of all enrolled patients) who obtained complete response (CR) with BV achieved long-term disease control without consolidative allogeneic transplant and may potentially be cured [6,7]. However, the impact of BV monotherapy on long-term survival outcomes remains unclear in cHL patients with early disease relapses after auto-SCT.

Fertility preservation and reproductive health are important issues for adolescents and young adult cancer survivors. The sequelae of premature loss of ovarian function can undoubtedly have undesirable effects for young women and girls, although the benefits of systemic anti-cancer therapies include sustained remission or cure. There is a lack of human data evaluating the effects of BV on pregnancy and the developing fetus, while animal studies have revealed evidence of caused embryofetal mortality and structural abnormalities. Thus, it is recommended that BV be avoided during or shortly before pregnancy.

Herein, we report a special clinical case of successful pregnancy and fetal outcome in a patient with cHL who achieved CR for six years with BV for early relapse defined as relapse within 12 months after auto-SCT. This case report was previously presented as a meeting poster at the 81st Annual Meeting of the Japanese Society of Hematology on October 11, 2019.

## Case presentation

**Figure 1:**
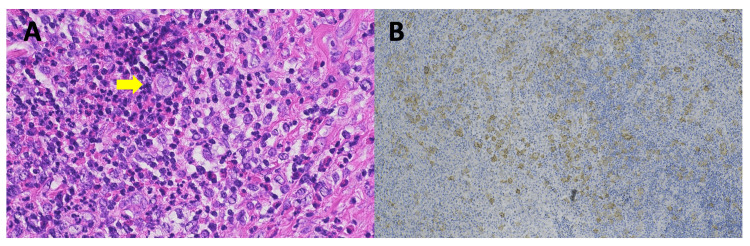
Histopathology and immunohistochemistry of the cervical lymph node at initial diagnosis. (A) Hematoxylin and eosin (H&E) staining, original magnification x400. H&E staining showed Hodgkin-Reed-Sternberg (HRS) cells (arrow) with lobulated nuclei and abundant cell bodies in clusters. (B) CD30 immunohistochemical staining, original magnification x100. HRS cells were positive for CD30.

A 27-year-old Japanese woman presented with a two-month history of swelling of the left supraclavicular lymph nodes in November 2014. A computed tomography (CT) scan revealed multiple enlarged lymph nodes in the cervical, supraclavicular, axillary, and mediastinum regions as well as soft tissue masses in the left pectoral muscle and the eighth thoracic vertebra. A lymph node biopsy confirmed the diagnosis of nodular sclerosis cHL (Figures 1A, 1B). A bone marrow biopsy showed no involvement. The clinical stage was ⅣA with multiple extralymphatic organ involvement according to the Ann Arbor classification. The patient preferred fertility preservation before initiating chemotherapy. She received buserelin acetate nasal spray in the cycle prior to egg retrieval. She underwent controlled ovarian stimulation following downregulation with a gonadotropin-releasing hormone agonist in a long protocol while receiving 10 mg of prednisolone daily to inhibit tumor growth. Human menopausal gonadotrophin injections were started on the third day of menstruation in the natural cycle. Transvaginal ultrasound-guided oocyte retrieval was performed. Thirteen oocytes were collected on an outpatient basis under local anesthesia. Nine of these oocytes had two pronuclei (2PN). The embryos obtained from intracytoplasmic sperm injection with her husband's sperm were cryopreserved. The patient had eight frozen embryos, including seven 8-cell embryos on day 3 and one 4CC grade (the Gardner score) embryo on day 7. The patient received a total of six cycles of the ABVD regimen (25 mg/m^2^ doxorubicin, 10 mg/m^2^ bleomycin, 6 mg/m^2^ vinblastine, and 375 mg/m^2^ dacarbazine; days 1 and 15, every four weeks). Interim fluorine-18 fluorodeoxyglucose (FDG) positron emission tomography/CT (PET/CT) scan after two cycles of chemotherapy demonstrated a complete metabolic response (CMR).

Three months after completion of the initial treatment, relapse occurred in the right cervical lymph nodes. The patient was then transferred to our institution. The laboratory findings on admission were normal lactate dehydrogenase; C-reaction protein, 1.05 mg/dL (normal range, <0.03 mg/dL); and soluble interleukin-2 receptor, 366 U/mL (normal range, 122-496 U/mL). A CT scan showed multiple lymph nodes in the right cervical, supraclavicular, and accessory nerve areas. A bone marrow biopsy showed no involvement. Biopsy and histopathologic findings of the cervical lymph node revealed recurrent cHL. The patient received CHASE (1,200 mg/m^2^ cyclophosphamide on day 1; 2,000 mg/m^2^ high-dose cytarabine from days 1 to 3; 40 mg/body dexamethasone from days 1 to 3; and 100 mg/m^2^ etoposide from days 1 to 3) regimen, which was the standard salvage chemotherapy at the time [8]. The disease remained stable after one cycle of chemotherapy. She then received one cycle of GDC (1,000 mg/m^2^ gemcitabine on days 1 and 8; 40 mg/body dexamethasone from days 1 to 4; and area under the blood concentration-time the curve (AUC) 5 carboplatin on day 1) as an effective and less-toxic salvage regimen with stable disease [9]. Subsequently, she underwent high-dose chemotherapy followed by auto-SCT with a conditioning regimen consisting of ranimustine (200 mg/m^2^ on days -8 and -3), carboplatin (300 mg/m^2^ from days -7 to -4), etoposide (500 mg/m^2^ from days -6 to -4), and cyclophosphamide (50 mg/kg from days -3 to -2) (MCEC regimen). Peripheral blood stem cells were mobilized with high-dose etoposide (500 mg/m^2^ from days 1 to 3) and granulocyte colony-stimulating factor. 

On day 7, the patient presented with pericardiac chest pain, shortness of breath, and dyspnea on exertion. A chest X-ray showed cardiomegaly and bilateral pleural effusions. An electrocardiogram revealed sinus tachycardia and low QRS voltage without ST-segment abnormalities. Transthoracic echocardiography showed an ejection fraction of 61%, a small pericardial effusion measuring 9 mm, no wall motion abnormalities, and normal diastolic function. The patient was diagnosed with acute pericarditis according to the 2015 European Society of Cardiology Guidelines/diagnostic criteria (pericardial chest pain and pericardial effusion) [10]. Continuous intravenous infusion of carperitide was started at a dose of 0.05 μg/kg/min and maintained at 0.08 μg/kg/min. The patient then changed to oral furosemide 20 mg and spironolactone 25 mg, resulting in a complete resolution of her symptoms. Neutrophil engraftment was achieved on day +8. A post-transplant CT scan revealed residual cervical and supraclavicular lymph nodes, and therefore, she received additional radiotherapy at a dose of 40 Gray in 20 fractions. PET/CT scan after radiotherapy showed a CMR. The patient received one cycle of combined estrogen-progesterone therapy (Kaufman therapy) one month after radiotherapy and got a natural menstrual cycle.

**Figure 2:**
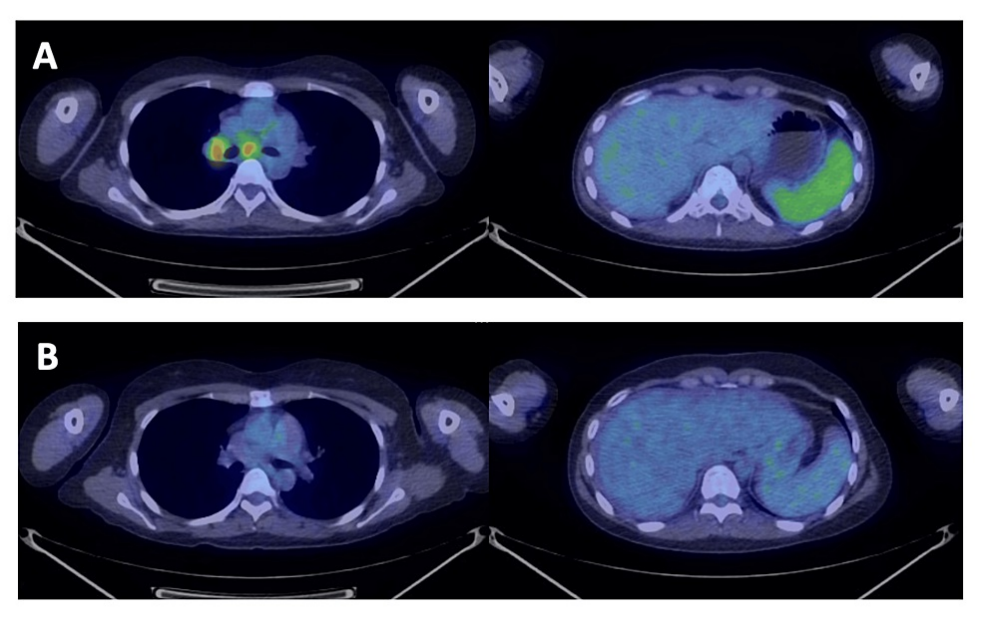
18F-FDG PET/CT. (A) A PET/CT scan revealed FDG uptake in the enlarged mediastinal and hilar lymph nodes and spleen. The patient relapsed six months after autologous stem cell transplant. (B) A PET/CT scan after 16 cycles of brentuximab vedotin demonstrated a complete metabolic response. 18F-FDG: fluorine-18 fluorodeoxyglucose, PET: positron emission tomography.

**Figure 3:**
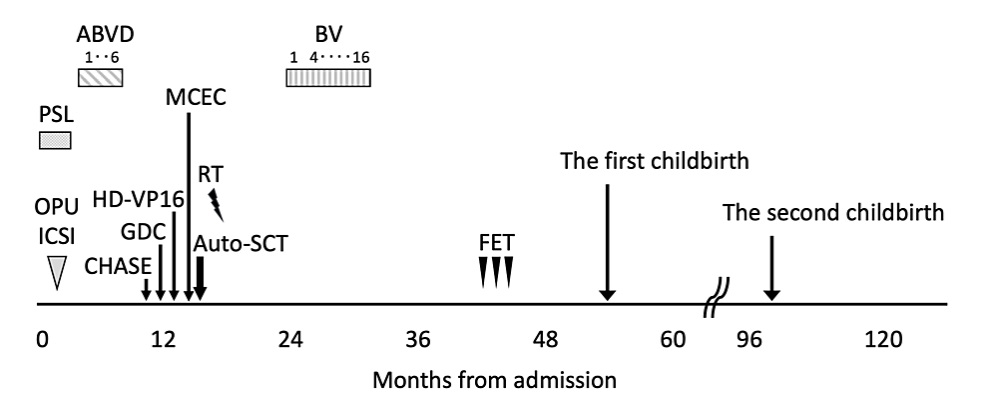
Clinical course. PSL: prednisolone, OPU: ovum pick up, ICSI: intracytoplasmic sperm injection, ABVD: doxorubicin, bleomycin, vinblastine, and dacarbazine regimen, CHASE: cyclophosphamide, high-dose cytarabine, dexamethasone, and etoposide regimen, GDC: gemcitabine, dexamethasone, and cisplatin regimen, HD-VP16: high-dose etoposide, MCEC: ranimustine, carboplatin, etoposide, and cyclophosphamide regimen, Auto-SCT: autologous stem cell transplant, RT: radiotherapy, BV: brentuximab vedotin, FET: frozen embryo transfer.

Six months after transplant, a follow-up enhanced CT scan showed the presence of enlarged mediastinal lymph nodes. A subsequent PET/CT scan revealed FDG uptake in the enlarged mediastinal and hilar lymph nodes and spleen, suggesting the recurrence of lymphoma (Figure 2). The patient received BV at 1.8 mg/kg on day 1 of each 21-day cycle for up to 16 cycles as salvage therapy after auto-SCT for relapsed cHL. After four cycles of BV, an enhanced CT scan showed a complete response, and the patient expressed a strong desire to plan a pregnancy in the near future. The hematology and reproductive departments repeatedly explained to the patient about the risk of lymphoma recurrence during pregnancy and early after childbirth and discussed future pregnancy plans. Considering that the patient had relapsed and refractory lymphoma, we decided to continue BV treatment for up to 16 cycles. She completed 16 cycles of BV and achieved a CMR on a PET/CT scan (Figures 2A, 2B). No adverse events including peripheral neuropathy were observed with BV treatment (Figure 3).

Six months after the BV completion, the patient expressed her strong desire to bear a child. We performed three cycles of timing therapy with the aim of achieving a natural pregnancy six months after completion of BV treatment, but no pregnancy occurred. The hormonal profile test revealed the following results: luteinizing hormone (LH), 113.7 mIU/mL (normal range, 1.76-10.24 mIU/mL in follicular phase of menstrual cycle); follicle-stimulating hormone (FSH), 153.9 mIU/mL (normal range, 3.01-14.72 mIU/mL in follicular phase of menstrual cycle); estradiol (E2), 10 pg/mL (normal range, 28.8-196.8 pg/mL in follicular phase of menstrual cycle); and anti-Müllerian hormone, <0.01 ng/mL (normal range at 30-32 years, 0.64-14.2 ng/mL). A diagnosis of premature ovarian failure was made, and she requested for frozen embryo transfer (FET). The patient underwent a standard pretreatment protocol of gonadotropin-release hormone agonist (buserelin acetate nasal spray) to inhibit ovulation, transdermal estradiol patches to prepare the endometrium, and progesterone vaginal suppositories to maintain implantation and pregnancy after FET. A single cleavage embryo was transferred due to the low number of fertilized oocytes. Blood tests including tumors markers were performed at the start of each menstrual cycle, and imaging tests were also performed at appropriate times. A pregnancy was successfully achieved through the third FET, 11 months after completing BV treatment. Her pregnancy course was free from adverse events. She had labor induced at 41 weeks and underwent cesarean section for non-reassured fetal status. She gave birth to a healthy baby. Two years after giving birth, she became pregnant again after FET. She did not conceive spontaneously. The pregnancy was uneventful, and she delivered a healthy baby by cesarean section, at 37 weeks. At the time of this writing, CMR has been maintained for six years after BV treatment.

## Discussion

Auto-SCT following high-dose chemotherapy has long been the standard care for patients with chemo-sensitive R/R cHL, showing significant improvement in failure-free survival, progression-free survival (PFS), and OS for patients with disease responding to second-line chemotherapy (60%, 62%, and 66%, respectively) compared to those who had a poor response (19%, 23%, and 17%, respectively) (p<0.001) [11]. Achievement of CR after pre-auto-SCT salvage chemotherapy is associated with improved clinical outcomes of post-auto-SCT. The most common first-line salvage regimens for R/R cHL are platinum-based and gemcitabine-based combination chemotherapy. There is no clear prospective evidence showing the superiority of the specific regimen [12].

The introduction of the novel agents BV and the checkpoint inhibitors nivolumab and pembrolizumab has transformed the management of R/R cHL, as these agents demonstrate notable activity in heavily pretreated patients. There has been ongoing interest in exploring the role of novel agents, especially in the context of pre-SCT salvage, post-auto-SCT consolidation, and the frontline treatment setting. Recent studies have shown evidence of the efficacy and safety of the combination of BV and chemotherapy or sequential BV-monotherapy followed by chemotherapy for patients with residual disease as salvage therapies prior to auto-SCT. In patients with R/R cHL, a multicenter phase Ⅰ/Ⅱ study demonstrated the efficacy and tolerability of BV combined with nivolumab as the first salvage therapy. The overall response rate and CR rates for all treated patients were 85% and 67%, with a three-year estimated PFS rate of 91% in patients who proceeded directly to transplant [13].

Historically, CHL patients who relapse after auto-SCT have had a poor prognosis. Several prognostic factors have been known to predict the risk of relapse after auto-SCT, including primary refractory disease, early relapse after frontline treatment, poor performance status, extranodal involvement, and PET-avoid residual disease prior to auto-SCT [14,15]. Recently, new therapeutic strategies have improved the survival outcomes of patients with cHL and early relapse after auto-SCT. The phase Ⅲ AETHERA trial demonstrated the efficacy of BV as consolidation therapy following auto-SCT in patients with cHL at a high risk of relapse or progression after auto-SCT. In the pivotal phase Ⅱ study of BV in patients with R/R cHL after failed auto-SCT, patients who achieved a CR had estimated five-year OS and PFS rates of 64% and 52%, respectively. Of the 13 patients who remained in CR at the time of study closure, nine patients (9% of all enrolled patients) remained in long-term remission without consolidative allogeneic transplant. Furthermore, patients who had a sustained CR tended to be younger with more extranodal disease and had a shorter interval from both initial diagnosis and most recent relapse to initiation of treatment with BV [14,15].

In our case, the patient had two adverse risk factors for relapse after auto-SCT, as defined in the AETHERA study, including relapse within 12 months from the end of frontline therapy and a history of primary refractory disease. For patients with two or more AETHERA risk factors, early consolidation with BV after auto-SCT significantly improves the PFS in patients with R/R cHL. Given that the use of BV as consolidation therapy after auto-SCT had not been approved in Japan at the time, the patient received BV due to relapse after auto-SCT. After completion of 16 courses of BV, the patient has remained in remission for more than six years without a consolidative allogeneic transplant and hence may be cured.

Chemotherapy-induced ovarian failure (COF) or premature menopause affects the quality of life of female cancer survivors. Previous studies have suggested that the mechanisms of COF may involve ovarian hypofunction by inducing oocyte apoptosis or disrupting granulosa cell function, causing depletion of primordial follicles, an increase in atretic follicles, and fibrosis of ovarian tissue [16]. Fertility preservation in cHL patients has gained increasing attention. In a retrospective cohort study, the number of pregnancies and births in young women treated with ABVD for cHL was similar to that of the general population. Although transient amenorrhea and premature menopause occurred more frequently in patients than in controls, fertility following ABVD was not affected [17]. However, the effects of BV on human fertility have not been evaluated. Based on findings in animal studies with MMAE-containing antibody-drug conjugates, BV may impair fertility in both women and men, whereas the effect on fertility is reversible after drug withdrawal. In ECHELON-1 (an open-label, randomized, phase Ⅲ study comparing BV with doxorubicin, vinblastine, and dacarbazine versus ABVD as frontline therapy for patients with advanced cHL), there was no significant difference in the number of pregnancies after either treatment in long-term follow-up [18]. Many organizations suggest that women postpone pregnancy for six to 12 months after finishing chemotherapy to prevent conception with an oocyte maturing during chemotherapy [19]. Patients must take precautions to prevent pregnancy during BV treatment and for at least six months after the last dose, as it is teratogenic based on animal studies.

## Conclusions

Novel targeted agents have improved survival and cure rates in patients with R/R cHL. This report describes a unique case of successful pregnancy and delivery in a cHL patient treated with BV for early relapse after auto-SCT, resulting in a CR for more than six years. Although this is a single case which limits the ability to generalize, BV may provide a potentially curative treatment for patients with cHL relapsed after auto-SCT. The recommended duration of contraception in female patients is at least six months after the last dose of BV. It is extremely important to discuss the risk of infertility and fertility preservation options with affected patients and collaborate with obstetricians and gynecologists prior to the initiation of cancer treatment.
